# Adult acute leukemia patients with gram-negative bacteria bloodstream infection: Risk factors and outcomes of antibiotic-resistant bacteria

**DOI:** 10.1007/s00277-024-05866-x

**Published:** 2024-07-03

**Authors:** Jinjin Wang, Mingchun Mu, Jinbing Zhu, Jinrong Yang, Yali Tao, Yuhui Chen, Qian Hu, Hui Zhou, Ailin Zhao, Ting Niu

**Affiliations:** 1 Department of Hematology, West China Hospital, Sichuan University, Chengdu, Sichuan China; 2 Gastric Cancer Center, West China Hospital, Sichuan University, Chengdu, Sichuan China

**Keywords:** Bloodstream infection, Gram-negative bacteria, Multidrug-resistant, Carbapenem-resistant, Risk factor, 30-day mortality

## Abstract

This study aims to analyze the risk factors for the development of multidrug-resistant (MDR) and carbapenem-resistant (CR) bacteria bloodstream infection (BSI) in a patient with acute leukemia (AL) and the mortality in gram-negative bacteria (GNB) BSI. This is a retrospective study conducted at West China Hospital of Sichuan University, which included patients diagnosed with AL and concomitant GNB BSI from 2016 to 2021. A total of 206 patients with GNB BSI in AL were included. The 30-day mortality rate for all patients was 26.2%, with rates of 25.8% for those with MDR GNB BSI and 59.1% for those with CR GNB BSI. Univariate and multivariate analyses revealed that exposure to quinolones (Odds ratio (OR) = 3.111, 95% confidence interval (95%CI): 1.623–5.964, p = 0.001) within the preceding 30 days was an independent risk factor for MDR GNB BSI, while placement of urinary catheter (OR = 6.311, 95%CI: 2.478–16.073, p < 0.001) and exposure to cephalosporins (OR = 2.340, 95%CI: 1.090–5.025, p = 0.029) and carbapenems (OR = 2.558, 95%CI: 1.190–5.497, p = 0.016) within the preceding 30 days were independently associated with CR GNB BSI. Additionally, CR GNB BSI (OR = 2.960, 95% CI: 1.016–8.624, p = 0.047), relapsed/refractory AL (OR = 3.035, 95% CI: 1.265–7.354, p = 0.013), septic shock (OR = 5.108, 95% CI: 1.794–14.547, p = 0.002), platelets < 30 × 10^9^/L before BSI (OR = 7.785, 95% CI: 2.055–29.492, p = 0.003), and inappropriate empiric antibiotic therapy (OR = 3.140, 95% CI: 1.171–8.417, p = 0.023) were independent risk factors for 30-day mortality in AL patients with GNB BSI. Prior antibiotic exposure was a significant factor in the occurrence of MDR GNB BSI and CR GNB BSI. CR GNB BSI increased the risk of mortality in AL patients with GNB BSI.

## Introduction

Bloodstream infection (BSI) refers to the isolation of at least one pathogen (bacteria and fungi) from blood samples. Patients with hematologic malignancies often develop BSI, with incidences ranging from 11 to 50%, and mortality rates ranging from 10 to 35.3% [1–4]. Acute leukemia (AL) is a common hematologic malignancy. Due to factors such as immunosuppression, bone marrow suppression, high-dose combination chemotherapy, and neutropenia, patients with AL often develop BSI during treatment, which can be life-threatening [5].

Recent studies showed that BSI caused by gram-negative bacteria (GNB) were more common than those caused by gram-positive bacteria [6, 7]. Our previous study also manifested that GNB BSI accounted for 70.4% in hematological malignancies [8]. According to epidemiological surveys in the United States, there were 279,000 cases of GNB BSI annually, with 41,900 deaths [9]. Moreover, the isolation of antibiotic-resistant strains in GNB BSI is increasing, leading to serious concerns about antimicrobial resistance. It is estimated that antimicrobial resistance causes 700,000 deaths globally each year, and this number was predicted to rise to 10 million by 2025 [10]. Several studies showed that the mortality rates of BSI in patients with hematologic malignancies caused by multidrug-resistant (MDR) bacteria and carbapenem-resistant (CR) bacteria were 20.2-33.3% and 20-74.1%, respectively [11–15]. Therefore, early diagnosis and timely effective treatment of BSI caused by antibiotic-resistant bacteria in AL patients are crucial. On the one hand, understanding the local prevalence patterns and antibiotic susceptibilities of pathogens helps clinicians choose appropriate antibiotic treatment. Studies revealed that inappropriate empiric antibiotic therapy was associated with poor prognosis in patients with hematologic malignancies who develop BSI [16, 17]. On the other hand, identifying risk factors for antibiotic-resistant bacterial BSI helps physicians recognize infections with antibiotic-resistant bacteria and intervene promptly to improve patient outcomes.

However, data on antibiotic-resistant GNB BSI in AL patients are limited, especially in the southwestern region of China. Therefore, our study aims to analyze local patterns of antibiotic-resistant GNB and antibiotic susceptibilities, evaluate risk factors associated with the 30-days mortality of GNB BSI, and identify risk factors associated with antibiotic-resistant bacterial BSI, in order to provide meaningful guidance for the prevention and treatment of antibiotic-resistant bacterial BSI in AL patients.

## Methods

### Setting and study design

This retrospective study was conducted at West China Hospital of Sichuan University, which has over 4300 beds. The subjects of this study were patients who were diagnosed with AL and blood cultures positive for GNB at the Department of Hematology, West China Hospital, from June 2016 to June 2021. The following conditions were excluded: (1) patients diagnosed with non-AL; (2) contaminated blood culture specimens; (3) incomplete clinical and laboratory data. The study was approved by the Ethics Committee of West China Hospital of Sichuan University.

### Data collection

The data were collected by the Hospital Information System (HIS) of West China Hospital of Sichuan University. The detail information was as follows: age, gender, underlying disease, treatment, hospital stays, complications, septic shock, laboratory results (neutrophil count, platelet count, pathogens of BSI, drug sensitivity test), invasive procedure (urinary catheter, central venous catheter (CVC), or peripherally inserted central catheter (PICC)), use of antibiotics, past 30 days of antibiotic exposure, clinical outcome (30 days after BSI). Data were extracted using a standardized data collection form and build an electronic database for analysis.

### Definitions

GNB BSI was defined as the presence of clinical manifestations of infection in a patient (such as fever, chills, tachycardia, or hypotension), along with the identification of GNB in blood cultures. MDR bacteria referred to bacteria resistant to three or more classes of antimicrobial agents. The definition of CR bacteria referred to isolates that exhibit intermediate or resistant to one or more carbapenems. An absolute neutrophil count of less than 0.5 × 10^9^ /L was defined as neutropenia, while less than 0.1 × 10^9^ /L was defined as profound neutropenia. Empirical antibiotic therapy referred to the administration of one or more antibiotics to suspected BSI patients within 48 h of blood culture collection and before obtaining drug sensitivity test. Appropriate empiric antibiotic therapy was defined as at least one of the empirically administered antibiotics demonstrates sensitivity in drug sensitive test; otherwise, it was deemed inappropriate empiric antibiotic therapy. Antibiotic exposure referred to patients receiving any intravenous or oral antibiotics for more than 48 h within the 30 days prior to the onset of BSI.

### Statistical analysis

Categorical variables were presented as frequencies and percentages, and between-group differences were compared using the Chi-square test or Fisher’s exact test. The median and interquartile range (IQR) were used to represent continuous data, while the t-test or Mann-Whitney U test was applied to compare differences between groups. Variables with *p* < 0.05 in univariate analysis were included in the multivariable logistic regression analysis to determine the risk factors for developing MDR GNB BSI, CR GNB BSI in patients with AL, as well as the risk factors for 30-day mortality in patients with GNB BSI. The Kaplan-Meier method was applied to plot survival curves, and the log-rank test was used to compare differences. *p* < 0.05 was regarded as statistically significant. All analyses were conducted in IBM SPSS 26.0.

## Results

### Patient characteristics

This study included a total of 206 patients with GNB BSI in AL, among which acute myeloid leukemia (AML) accounted for 68.4% (141/206), and acute lymphoblastic leukemia (ALL) accounted for 31.6% (65/206). Among the 206 patients with AL, 74 (35.9%) had a disease status of relapsed or refractory. The median age of all patients was 41 (IQR, 27.8–54.0) years, with 84 (40.8%) being female. The median length of stay before BSI was 20 (IQR, 13.0-37.3) days, and hospital-acquired infection was observed in 194 (94.2%) patients. The majority of patients number (85.4%) developed pulmonary infections, 31 (15.0%) developed perianal infections, and 15 (7.3%) developed gastroenteritis. Additionally, 43 (20.9%) patients experienced septic shock. Before the onset of BSI, 177/206 (85.9%) patients had neutropenia, with 152/206 (73.8%) being profound neutropenia. Patients with urinary catheters and PICC/CVC were 26 (12.6%) and 107 (51.9%), respectively. Of the 206 patients, 58 (28.2%) received corticosteroid therapy. In all patients, 48 (23.3%) received inappropriate empiric antibiotic therapy at the onset of BSI, while 158 (76.7%) received appropriate empiric antibiotic therapy, with 41(19.9%) receiving monotherapy and 117 (56.8%) receiving combination therapy. General characteristics of the above patients are detailed in Table 1.

**Table 1:** Characteristics of GNB BSI patients stratified by outcome

Characteristic	All patients (*n* = 206)	Non-survivors (*n* = 54)	Survivors (*n* = 152)	*P* Value
Age, y				
Median (IQR)	41.0 (27.8, 54.0)	40.5 (26.0, 61.0)	41.0 (28.0, 53.0)	0.570
≥55	50 (24.3)	16 (29.6)	34 (22.4)	0.285
Female sex	84 (40.8)	12 (22.2)	72 (47.4)	**0.001**
Length of stay before BSI, d, median (IQR)	20.0 (13.0, 37.3)	30.0 (15.8, 48.8)	18.5 (13.0, 28.0)	**0.004**
Hospital-acquired infection	194 (94.2)	51 (94.4)	143 (94.1)	1.000
BSI due to drug-resistant bacteria				
BSI due to MDR strain	128 (62.1)	33 (61.1)	95 (62.5)	0.857
BSI due to CR strain	44 (21.4)	26 (59.1)	18 (40.9)	**<0.001**
Underlying diagnosis				0.091
AML	141 (68.4)	32 (59.3	109 (77.3)	
ALL	65 (31.6)	22 (40.7)	43 (28.3)	
Relapsed/refractory AL	74 (35.9)	31 (57.4)	43 (28.3)	**<0.001**
Hematopoietic stem cell transplantation	15 (7.3)	4 (7.4)	11 (7.2)	1.000
Complications				
Pulmonary infection	176 (85.4)	52 (96.3)	124 (81.6)	**0.008**
Gastroenteritis	15 (7.3)	1 (1.9)	14 (9.2)	0.138
Perianal infection	31 (15.0)	10 (18.5)	21 (13.8)	0.406
Septic shock	43 (20.9)	25 (46.3)	18 (11.8)	**<0.001**
Neutropenia before BSI	177 (85.9)	47 (87)	130 (85.5)	0.784
Profound neutropenia before BSI	152 (73.8)	35 (64.8)	117 (77)	0.081
Duration of neutropenia before BSI, d, median (IQR)	6.0 (2.0, 13.0)	10.0 (3.0, 23.0)	6.0 (2.0, 10.0)	**0.004**
Platelets < 30 × 10^9^/L before BSI	144 (69.9)	50 (92.6)	94 (61.8)	**<0.001**
Placement of urinary catheter	26 (12.6)	16 (29.6)	10 (6.6)	**<0.001**
Placement of PICC/CVC	107 (51.9)	23 (42.6)	84 (55.3)	0.109
Inappropriate empiric antibiotic treatment	48 (23.3)	23 (42.6)	25 (16.4)	**<0.001**
Appropriate empiric antibiotic treatment				0.065
Monotherapy	41 (25.9)	4 (12.9)	37 (29.1)	
Combination	117 (74.1)	27 (87.1)	90 (70.9)	
Corticosteroid use	58 (28.2)	20 (37)	38 (25)	0.091

### Microbiology

**Fig. 1:**
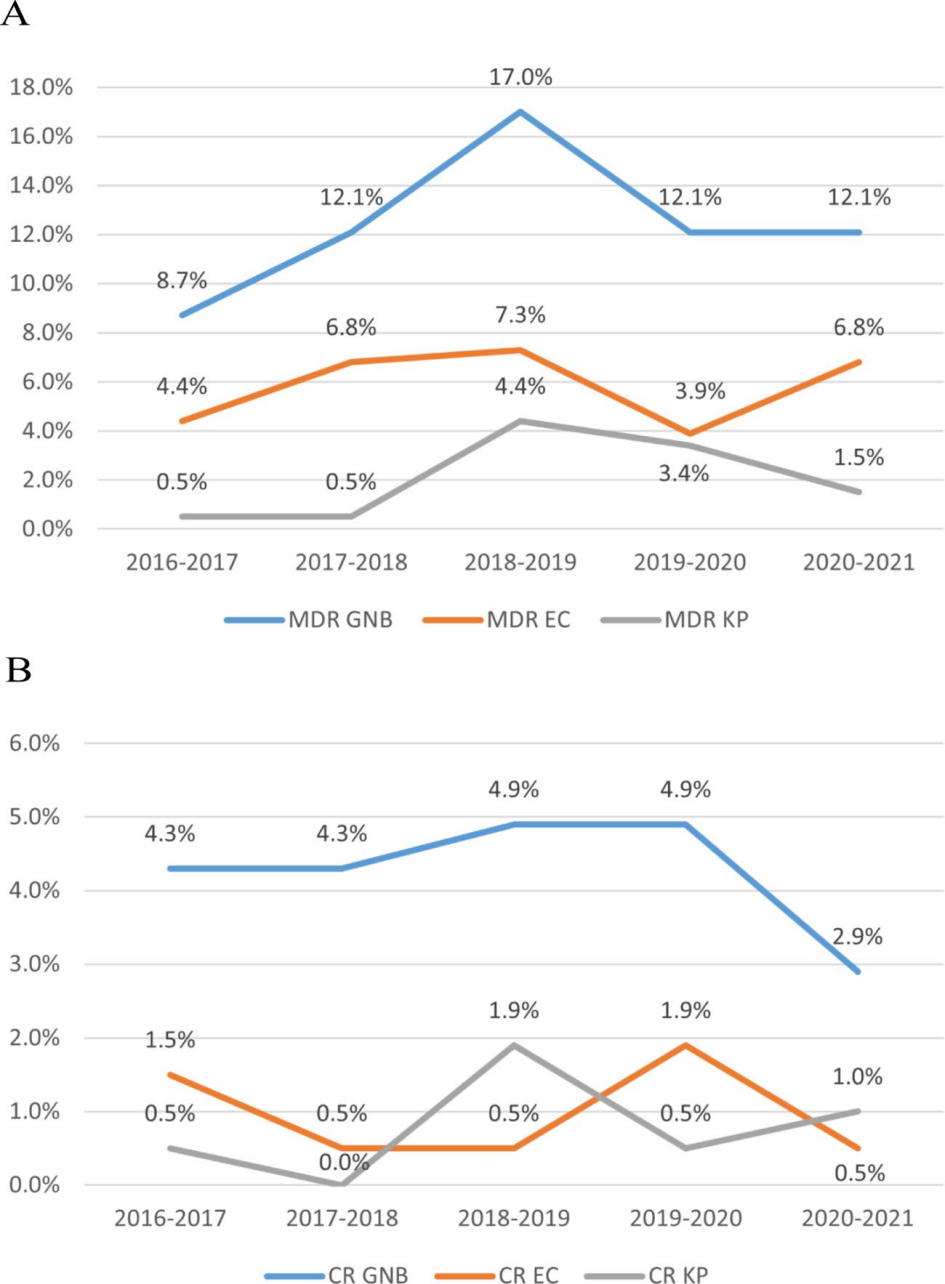
Trend in MDR (**A**) and CR (**B**) strains of BSI in AL patients. EC: *Escherichia coli*; KP: *Klebsiella pneumoniae*

Table 2 shows the common GNB and their antibiotic resistance characteristics. A total of 206 GNB were isolated, with *Escherichia coli* (*E. coli)* (81/206, 39.3%), *Klebsiella pneumoniae* (*K. pneumoniae)* (46/206, 22.3%), and *Pseudomonas aeruginosa* (*P. aeruginosa)* (34/206, 16.5%) being the most common. Among all GNB, MDR GNB accounted for 62.1% (128/206), most commonly observed in *E*. *coli* (46.9%), followed by *P. aeruginosa* (22.7%) and *K. pneumoniae* (16.4%). Forty-four strains (21.4%) were identified as CR bacteria, mainly found in *E*. *coli* (22.7%), *P. aeruginosa* (18.2%), and *K*. *pneumoniae* (18.2%). Five strains of *A. baumannii* were isolated in the study, all of which were MDR and CR. Overall, as shown in Fig. 1, there was a trend of initial increase followed by decrease in MDR GNB and CR GNB from 2016 to 2021. MDR *K*. *pneumoniae* also showed a similar trend of increase followed by decrease over the five years, while MDR *E*. *coli* and CR *K*. *pneumoniae* displayed an increasing trend from 2019 to 2021.

**Table 2:** Antimicrobial resistance profiles of all GNB and isolated MDR and CR strains

Microorganisms	Total											MDR isolates	CR isolates
		Piperacillin/tazobactam	Cefoperazone subactam	Ceftriaxone	Cefepime	Ceftazidine	Amikacin	Gentamicin	Ciprofloxacin	Meropenem	Imipenem		
Total	206	66 (32.0)	63 (30.6)	110 (53.4)	55 (26.7)	44 (21.4)	15 (7.3)	52 (25.2)	88 (42.8)	33 (16.1)	43 (20.9)	128 (62.1)	44 (21.4)
*E. coli*	81	23 (28.4)	24 (29.6)	55 (67.9)	27 (33.3)	21 (25.9)	4 (4.9)	27 (33.3)	60 (74.1)	6 (7.4)	7 (8.6)	60 (74.1)	10 (12.3)
*K. pneumoniae*	46	20 (43.5)	19 (41.3)	16 (34.8)	13 (28.3)	12 (26.1)	1 (2.2)	9 (19.6)	14 (30.4)	8 (17.4)	8 (17.4)	21 (45.7)	8 (17.4)
*P. aeruginosa*	34	8 (23.5)	8 (23.5)	23 (67.6)	4 (11.8)	3 (8.8)	1 (2.9)	2 (5.9)	1 (2.9)	7 (20.6)	10 (29.4)	29 (85.3)	8 (23.5)
*S. maltophilia*	8	—	—	—	—	—	—	—	—	—	—	2 (25)	2 (25)
*E. cloacae*	7	3 (42.9)	—	2 (28.6)	1 (14.3)	1 (14.3)	0	2 (28.6)	1 (14.3)	1 (14.3)	2 (28.6)	1 (14.3)	1 (14.3)
*A. baumannii*	5	5 (100)	5(100.0)	5 (100.0)	5 (100.0)	—	2 (40.0)	4 (80.0)	5 (100.0)	4 (80.0)	5 (100.0)	5 (100.0)	5 (100.0)
Others	25	—	—	10 (40.0)	5 (20.0)	—	4 (16.0)	6 (24.0)	4 (16.0)	8 (32.0)	8 (32.0)	10 (40.0)	10 (40.0)
MDR isolates	128	56 (43.8)	74 (57.8)	96 (75.0)	51 (39.8)	72 (56.3)	15 (11.7)	49 (38.3)	78 (60.9)	45 (35.2)	39 (30.5)	—	—
CR isolates	44	32 (72.7)	34 (77.3)	30 (68.2)	26 (59.1)	32 (72.7)	11 (25.0)	20 (45.5)	24 (54.5)	38 (86.4)	39 (88.6)	—	—

As shown in Table 2, all GNB showed relatively low resistance to amikacin (7.3%), meropenem (16.1%), and imipenem (20.9%). In common GNB, *E*. *coli* and *K*. *pneumoniae* exhibited low resistance to amikacin (4.9%, 2.2%), meropenem (7.4%, 17.4%), and imipenem (8.6%, 17.4%). While *P. aeruginosa* had low resistance to amikacin (2.9%), ciprofloxacin (2.9%), and gentamicin (5.9%). All *A. baumannii* isolates were completely resistant to most antibiotics (piperacillin/tazobactam, cefoperazone subactam, ceftriaxone, cefepime, ciprofloxacin, and imipenem). Overall, MDR and CR bacteria exhibited relatively high resistance to most antibiotics, except for amikacin (11.7%, 25.0%).

### Risk factors for MDR GNB BSI and CR GNB BSI

Univariate analysis showed that female sex, placement of PICC/CVC, and exposure to carbapenems/aminoglycosides/quinolones within the preceding 30 days were potential risk factors for MDR GNB BSI in AL patients (Table 3). Further multivariate analysis revealed that exposure to quinolones within the preceding 30 days (Odds ratio (OR) = 3.111, 95% confidence interval (95%CI): 1.623–5.964, *p* = 0.001) was an independent risk factor for MDR GNB BSI in AL patients (Table 4). Similarly, differences were found between CR GNB BSI and Non-CR GNB BSI in univariate analysis for factors such as placement of urinary catheter and exposure to cephalosporins and carbapenems within the preceding 30 days (Table 3). Multivariable logistic regression analysis indicated that placement of urinary catheter (OR = 6.311, 95%CI: 2.478–16.073, *p* < 0.001), exposure to cephalosporins (OR = 2.340, 95%CI: 1.090–5.025, *p* = 0.029), and carbapenems (OR = 2.558, 95%CI: 1.190–5.497, *p* = 0.016) within the preceding 30 days were independently associated with CR GNB BSI in AL patients (Table 4). Furthermore, this study also showed that the 30-day mortality rate was significantly higher in AL patients with CR GNB BSI (59.1%) compared to those with Non-CR GNB BSI (17.3%) (*p* < 0.001), while there was no difference between MDR GNB BSI (25.8%) and Non-MDR GNB BSI (26.9%) (*p* = 0.857) (Table 3).

**Table 3:** Univariate analysis of factors associated with BSI caused by MDR and CR GNB

Characteristic	MDR (*n* = 128)	Non-MDR (*n* = 78)	*P* value		CR (*n* = 44)	Non-CR(*n* = 162)	*P* value
Age, y, median (IQR)	41.5 (28.3, 54.8)	39.0 (24.0, 53.3)	0.207		40.5 (28.3, 52.0)	41.0 (26.0, 55.3)	0.855
Female sex	45 (35.2)	39 (50.0)	**0.035**		14 (31.8)	70 (43.2)	0.173
Length of stay before BSI, d, median (IQR)	21.0 (13.3, 38.8)	19.0 (12.8, 30.8)	0.416		27.5 (10.8, 45.5)	19.0 (13.0, 31.3)	0.156
Underlying diagnosis			0.096				0.254
AML	93 (72.7)	48 (61.5)			27 (61.4)	114 (70.4)	
ALL	35 (27.3)	30 (38.5)			17 (38.6)	48 (29.6)	
Relapsed/refractory acute leukemia	41 (32.0)	33 (42.3)	0.136		16 (36.4)	58 (35.8)	0.945
Hematopoietic stem cell transplantation	11 (8.6)	4 (5.1)	0.353		3 (6.8)	12 (7.4)	0.894
Neutropenia before BSI	110 (85.9)	67 (85.9)	0.994		38 (86.4)	139 (85.8)	0.924
Profound neutropenia before BSI	92 (71.9)	60 (76.9)	0.424		28 (63.6)	124 (76.5)	0.084
Duration of neutropenia before BSI, d, median (IQR)	6.0 (2.0, 15.8)	6.0 (2.0, 10.8)	0.250		7.0 (2.3, 19.0)	6.0 (2.0, 13.0)	0.344
Platelets < 30 × 10^9^/L before BSI	93 (72.7)	51 (65.4)	0.270		36 (81.8)	108 (66.7)	0.052
Placement of urinary catheter	19 (14.8)	7 (9.0)	0.219		13 (29.5)	13 (8.0)	**<0.001**
Placement of PICC/CVC	74 (57.8)	33 (42.3)	**0.031**		22 (50.0)	85 (52.5)	0.771
Corticosteroid use	32 (25.0)	26 (33.3)	0.197		16 (36.4)	42 (25.9)	0.172
Antibiotic exposure							
Penicillins	45 (35.2)	31 (39.7)	0.508		16 (36.4)	60 (37.0)	0.935
Cephalosporins	62 (48.4)	27 (34.6)	0.052		26 (59.1)	63 (38.9)	**0.016**
Carbapenems	69 (53.9)	30 (38.5)	**0.031**		30 (68.2)	69 (42.6)	**0.003**
Aminoglycosides	13 (10.2)	2 (2.6)	**0.042**		5 (11.4)	10 (6.2)	0.396
Quinolones	57 (44.5)	16 (20.5)	**<0.001**		20 (45.5)	53 (32.7)	0.117
30-d mortality	33 (25.8)	21 (26.9)	0.857		26 (59.1)	28 (17.3)	**<0.001**

**Table 4:** Multivariate analysis of risk factors for MDR and CR GNB BSI, as well as 30-day mortality risk factors in GNB BSI patients

Variable	OR	(95% CI)	*P* Value
**MDR GNB BSI**			
Exposure to quinolones	3.111	(1.623–5.964)	0.001
**CR GNB BSI**			
Placement of urinary catheter	6.311	(2.478–16.073)	<0.001
Exposure to cephalosporins	2.340	(1.090–5.025)	0.029
Exposure to carbapenems	2.558	(1.190–5.497)	0.016
**30-day mortality of GNB BSI**			
BSI due to CR strain	2.960	(1.016–8.624)	0.047
Relapsed/refractory acute leukemia	3.035	(1.265–7.354)	0.013
Septic shock	5.108	(1.794–14.547)	0.002
Platelets < 30 × 10^9^/L before BSI	7.785	(2.055–29.492)	0.003
Inappropriate empiric antibiotic treatment	3.140	(1.171–8.417)	0.023

### Outcome of GNB BSI

**Fig. 2:**
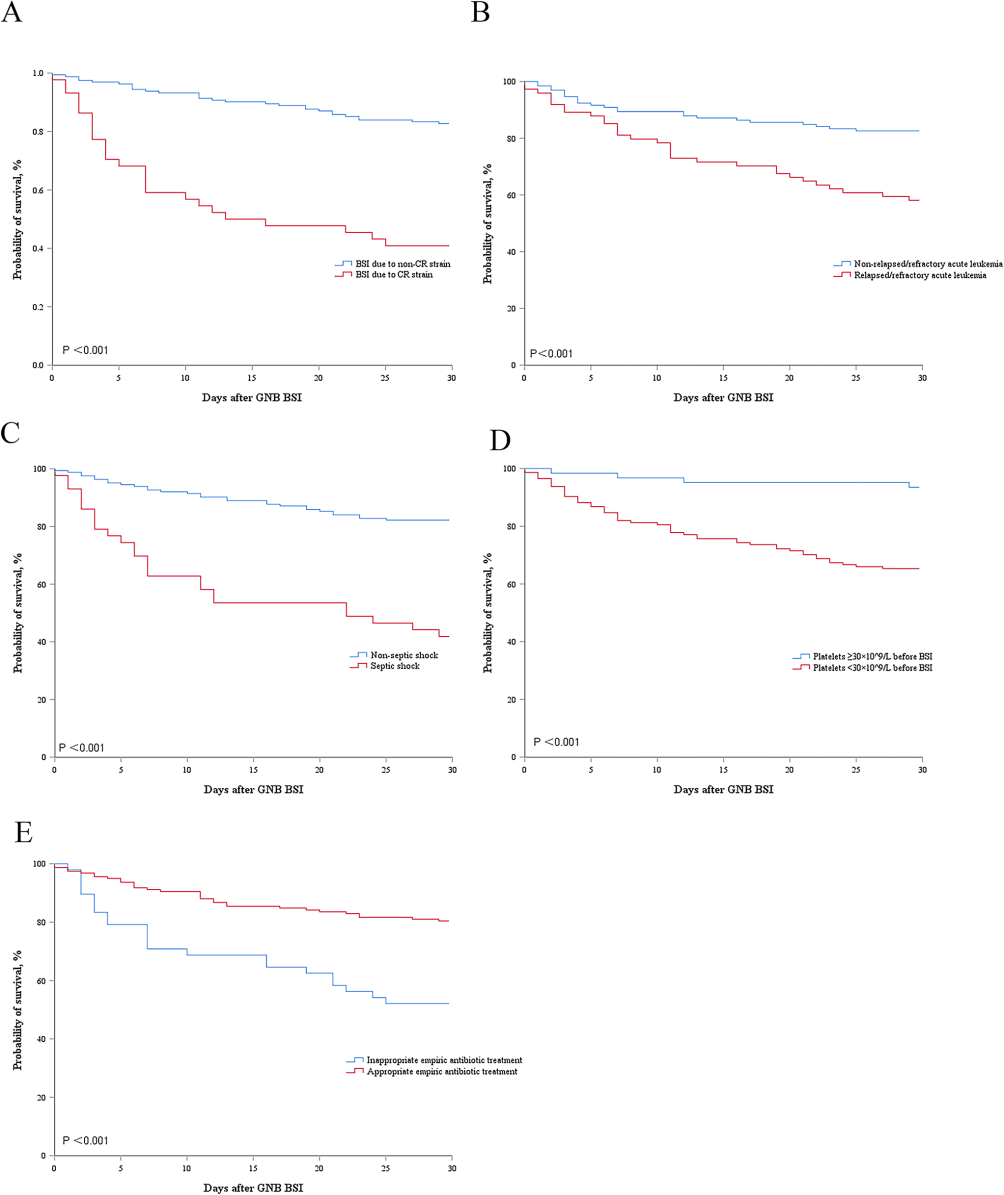
Kaplan-Meier curves of the estimated 30-day probability of survival for GNB BSI caused by CR strain and non–CR strain (**A**); patients with and without relapsed/refractory AL (**B**); patients with and without septic shock (**C**); patients with and without platelets < 30 × 10^9^/L before BSI (**D**); and patients with and without inappropriate empiric treatment (**E**)

This study showed that the 30-day mortality rate among AL patients with GNB BSI was 26.2%. We analyzed the risk factors for 30-day mortality in AL patients with GNB BSI, and the results revealed differences between survivors and non-survivors in the following factors: female sex, length of stay before BSI, CR GNB BSI, relapsed/refractory AL, pulmonary infection, septic shock, duration of neutropenia before BSI, platelets < 30 × 10^9^/L before BSI, and inappropriate empiric antibiotic therapy (Table 1). The above-mentioned variables were included in the multivariate analysis, the results indicated that CR GNB BSI (OR = 2.960, 95% CI: 1.016–8.624, *p* = 0.047), relapsed/refractory AL (OR = 3.035, 95% CI: 1.265–7.354, *p* = 0.013), septic shock (OR = 5.108, 95% CI: 1.794–14.547, *p* = 0.002), platelets < 30 × 10^9^/L before BSI (OR = 7.785, 95% CI: 2.055–29.492, *p* = 0.003), and inappropriate empiric antibiotic therapy (OR = 3.140, 95% CI: 1.171–8.417, *p* = 0.023) were independent risk factors for 30-day mortality in AL patients with GNB BSI (Table 4). Survival analysis was conducted on AL patients with GNB BSI, revealing that CR GNB BSI (*p* < 0.001), relapsed/ refractory AL (*p* < 0.001), septic shock (*p* < 0.001), platelets < 30 × 10^9^/L before BSI (*p* < 0.001), and inappropriate empiric antibiotic therapy (*p* < 0.001) were associated with poorer 30-day survival rates (Fig. 2).

## Discussion

With the widespread use of antibiotics, bacteria have developed various mechanisms, such as producing inactivating enzymes (carbapenemases), mutations in target sites, and reduced bacterial outer membrane permeability [18]. Antimicrobial resistance has become a global concern. However, data on the epidemiology and prognosis of BSI caused by drug-resistant bacteria in patients with hematologic malignancies are limited [11–13], especially in AL patients. Our study revealed that among AL patients with GNB BSI, the proportion of antibiotic-resistant bacterial BSI was considerable, with MDR GNB BSI accounting for 62.1% and CR GNB BSI accounting for 21.4%. Moreover, our hospital data showed that previous antibiotic exposure within 30 days was a significant factor associated with the occurrence of MDR GNB BSI and CR GNB BSI. This underscores the importance of judicious antibiotic use to avoid overuse. The 30-day mortality rate of AL patients with GNB BSI in our hospital was 26.2%, consistent with other studies (21.6-28.6%) [13, 19, 20]. However, the mortality rate of antibiotic-resistant bacteria BSI, especially CR GNB BSI, was high, with a 30-day mortality rate of 59.1%. Previous studies have also shown mortality rates for CR bacterial BSI ranging from 42.5 to 74.1% [13, 15, 21]. This highlights the significant threat of antibiotic-resistant bacterial BSI to patient life, necessitating timely identification of pathogens, appropriate antibiotic treatment, and regular hospital surveillance to improve patient management and prognosis.

In this study, GNB causing BSI was most commonly *E*. *coli*, followed by *K*. *pneumoniae* and *P. aeruginosa*, consistent with reports from China, Italy, and Brazil [6, 13, 22]. However, a retrospective study in Saudi Arabia showed that GNB BSI in AL patients was more commonly caused by *K*. *pneumoniae* (33.3%) [23]. This indicates regional differences in the prevalence patterns of pathogens, highlighting the importance of ongoing local pathogen surveillance. Our study found that isolated GNB exhibited varying degrees of resistance to commonly used antibiotics. *E*. *coli* and *K*. *pneumoniae* showed low resistance to amikacin and carbapenems (imipenem and meropenem), while *P. aeruginosa* exhibited low resistance to amikacin and quinolones. Therefore, the above appropriate antibiotics can be selected for clinical infections caused by these bacteria.

Antibiotic-resistant bacteria were a major focus of our study. Antibiotic-resistant bacterial infections not only lead to poor prognosis but also increase patient hospitalization time and economic burden. Wang et al. found that MDR bacterial BSI accounted for 57% in AML patients [24], while Schonardie et al. showed that CR bacterial BSI accounted for 17.3% of BSI in patients with hematologic malignancies [13]. Our study revealed that MDR GNB BSI accounted for 62.1% and CR GNB BSI accounted for 21.4% of all BSI in AL patient. The monitoring system of the China Antimicrobial Surveillance Network (CHINET) in 2021 showed a continuous decline in the detection rates of various important CR bacteria in recent years, despite their increasing trend over several years [25]. Our study also found a trend of initially increasing and then decreasing MDR GNB and CR GNB from 2016 to 2021, possibly due to the attention given by the hematologists in our hospital to antibiotic-resistant bacteria and the proper use of antibiotics. However, because of antibiotic overuse, resistant bacterial infections have become a significant threat to patient health, necessitating the development of new antibiotics targeting resistant bacteria [26].

CR GNB BSI was associated with poor prognosis, with a previous study showing an increased risk of death in patients with CR bacterial BSI [27]. Our study similarly found CR GNB BSI to be one of the independent risk factors for 30-day mortality in AL patients. Although MDR GNB BSI did not increase the risk of death in our study, another study showed it to be a risk factor for mortality [12]. This difference may be due to sample size and regional variations.

As antibiotic-resistant bacterial BSI is closely related to prognosis, our study analyzed the risk factors for the occurrence of antibiotic-resistant bacterial BSI. Our study demonstrated that prior use of quinolone antibiotics within the past 30 days was a risk factor for developing MDR GNB BSI. Studies by Tacconelli et al. and Zhao et al. also showed that exposure to quinolones was associated with MDR bacterial BSI [12, 28]. As shown in previous studies, exposure to carbapenems in the past was independently associated with CR bacterial BSI [12, 29]. Our study also confirmed this result. Therefore, clinicians need to use antibiotics cautiously to prevent misuse. Clinicians can avoid overuse of these antibiotics by using a few available antibiotics more judiciously to prevent the emergence of more antibiotic-resistant bacteria. In addition, our study found that placement of urinary catheter and exposure to cephalosporins within 30 days were independent risk factors for CR GNB BSI. A multicenter retrospective study verified a significantly higher proportion of placement of urinary catheter developing CR bacterial infections [30].

Antibiotics are indispensable in the treatment of BSI, and timely and effective empiric antibiotic therapy is key to controlling BSI. Ricard et al. found that delayed antibiotic use was associated with increased in-hospital mortality in septic patients [31]. A series of studies emphasized that inappropriate empiric antibiotic therapy was a risk factor for death in patients with hematologic malignancies [16, 32, 33]. In our study, 23.3% of patients received inappropriate empiric antibiotic therapy, and we found that inappropriate empiric antibiotic therapy was one of the independent risk factors for 30-day mortality in AL patients with GNB BSI. Therefore, antibiotic therapy is crucial for AL patients with BSI. Understanding the local distribution of pathogens and antibiotic susceptibility is helpful for clinicians to choose appropriate antibiotics.

In addition to CR GNB BSI and inappropriate empiric antibiotic therapy being associated with 30-day mortality in AL patients with GNB BSI, our study and other studies confirmed that relapsed/refractory AL and septic shock were risk factors for 30-day mortality in patients with GNB BSI [21, 22]. Therefore, patients with clinical manifestations such as hypotension, low oxygen saturation, and altered consciousness need to be identified early and provided with timely and effective interventions to restore circulation through fluid resuscitation, vasopressors, and other treatments. We also found that platelets < 30 × 10^9^/L before BSI were associated with 30-day mortality. Besides its role in hemostasis, platelets also participate in preventing bacterial infections. Platelets can recognize microbial antigens and enhance immune responses, thereby preventing bacterial infections [34].

BSI is common in patient with AML or ALL. Previous studies have shown that the type of AL (AML or ALL) was not an independent risk factor for antibiotic-resistant bacterial BSI or mortality of BSI in patients with hematologic diseases [12, 13, 35]. Similarly, our study did not find that the subtype of AL was a risk factor for 30-day mortality in BSI or for the occurrence of MDR and CR GNB BSI. In contrast, Micozzi et al. showed that AML was an independent risk factor for the occurrence of BSI in hematologic malignancy patients colonized with CR *K*. *pneumoniae* [36]. These conflicting conclusions may stem from the fact that most of current studies are retrospective, their conclusions may be limited by small sample sizes, with possible bias. Therefore, whether the subtype of AL affects the occurrence and prognosis of BSI is unclear, which requires further evaluation through larger, multicenter, and prospective studies. In our study, we did not find that neutropenia was an independent risk factor for the occurrence of antibiotic-resistant bacterial BSI or 30-day mortality in BSI patients. Currently, there is no consensus on whether neutropenia affects the prognosis of BSI patients with hematological diseases. Some studies indicated that neutropenia was unrelated to mortality in patients with hematologic diseases who develop BSI [12, 37, 38], while others demonstrated that the neutropenia or the duration of neutropenia impacted the prognosis of these patients [39–41]. Additionally, previous research showed that neutropenia was not significantly associated with the occurrence of CR BSI [13, 35]. Therefore, larger prospective studies are needed to investigate whether neutropenia affects the occurrence and prognosis of BSI in patients with hematologic diseases.

This study has some limitations, primarily due to its single-center retrospective nature and the limited number of collected BSI patients. Since the epidemiology of BSI varies by region, the conclusions of this study may not apply to centers with significant regional differences, and these limitations may also result in discrepancies with findings from other studies.

In conclusion, our study confirmed that the incidence of antibiotic-resistant bacterial BSI was not low among AL patients, and the mortality rate was high for CR GNB BSI. Prior antibiotic exposure was one of the independent risk factors for developing MDR GNB BSI and CR GNB BSI. Furthermore, CR GNB BSI increased the risk of mortality in AL patients with GNB BSI. Prospective studies are warranted to better understand the characteristics of antibiotic-resistant bacterial BSI in AL.

## Data Availability

The data presented in this study are available on request from the corresponding author.

## References

[1] Trecarichi EM, Pagano L, Candoni A et al (2015) Current epidemiology and antimicrobial resistance data for bacterial bloodstream infections in patients with hematologic malignancies: an Italian multicentre prospective survey. Clin Microbiol Infect 21:337–343. 10.1016/j.cmi.2014.11.022 25595706 10.1016/j.cmi.2014.11.022

[2] Nørgaard M, Larsson H, Pedersen G, Schønheyder HC, Sørensen HT (2006) Risk of bacteraemia and mortality in patients with haematological malignancies. Clin Microbiol Infect 12:217–223. 10.1111/j.1469-0691.2005.01298.x 16451407 10.1111/j.1469-0691.2005.01298.x

[3] Åttman E, Aittoniemi J, Sinisalo M et al (2015) Etiology, clinical course and outcome of healthcare-associated bloodstream infections in patients with hematological malignancies: a retrospective study of 350 patients in a Finnish tertiary care hospital. Leuk Lymphoma 56:3370–3377. 10.3109/10428194.2015.1032967 10.3109/10428194.2015.1032967 25813080

[4] Kolonen A, Sinisalo M, Huttunen R et al (2017) Bloodstream infections in acute myeloid leukemia patients treated according to the Finnish Leukemia Group AML-2003 protocol - a prospective nationwide study. Infect Dis (Lond) 49:799–808. 10.1080/23744235.2017.1347814 28683646 10.1080/23744235.2017.1347814

[5] Kara Ö, Zarakolu P, Aşçioğlu S et al (2015) Epidemiology and emerging resistance in bacterial bloodstream infections in patients with hematologic malignancies. Infect Dis (Lond) 47:686–693. 10.3109/23744235.2015.1051105 26024284 10.3109/23744235.2015.1051105

[6] Trecarichi EM, Giuliano G, Cattaneo C et al (2023) Bloodstream infections due to Gram-negative bacteria in patients with hematologic malignancies: updated epidemiology and risk factors for multidrug-resistant strains in an Italian perspective survey. Int J Antimicrob Agents 61:106806. 10.1016/j.ijantimicag.2023.106806 37030470 10.1016/j.ijantimicag.2023.106806

[7] Haddad S, Jabbour JF, Hindy JR et al (2021) Bacterial bloodstream infections and patterns of resistance in patients with haematological malignancies at a tertiary centre in Lebanon over 10 years. J Glob Antimicrob Resist 27:228–235. 10.1016/j.jgar.2021.09.008 34607062 10.1016/j.jgar.2021.09.008

[8] Wang J, Wang M, Zhao A et al (2023) Microbiology and prognostic prediction model of bloodstream infection in patients with hematological malignancies. Front Cell Infect Microbiol 13:1167638. 10.3389/fcimb.2023.1167638 37457950 10.3389/fcimb.2023.1167638 PMC10347389

[9] Al-Hasan MN (2020) Gram-negative bloodstream infection: implications of Antimicrobial Resistance on Clinical outcomes and Therapy. Antibiotics (Basel). 10.3390/antibiotics9120922 10.3390/antibiotics9120922 PMC7767175 33352973

[10] Huh K, Chung DR, Ha YE et al (2020) Impact of difficult-to-treat resistance in Gram-negative bacteremia on mortality: Retrospective Analysis of Nationwide Surveillance Data. Clin Infect Dis 71:e487. 10.1093/cid/ciaa084 31994704 10.1093/cid/ciaa084

[11] Amanati A, Sajedianfard S, Khajeh S et al (2021) Bloodstream infections in adult patients with malignancy, epidemiology, microbiology, and risk factors associated with mortality and multi-drug resistance. BMC Infect Dis 21:636. 10.1186/s12879-021-06243-z 34215207 10.1186/s12879-021-06243-z PMC8254331

[12] Zhao Y, Lin Q, Liu L et al (2020) Risk factors and outcomes of antibiotic-resistant Pseudomonas aeruginosa Bloodstream infection in adult patients with Acute Leukemia. Clin Infect Dis 71:S386. 10.1093/cid/ciaa1522 33367574 10.1093/cid/ciaa1522

[13] Schonardie AP, Beck E, Rigatto MH (2023) Prevalence of bloodstream infection pathogens in hemato-oncological patients and predictors of carbapenem-resistant gram-negative bacterial infections during febrile neutropenia. Braz J Infect Dis 27:102758. 10.1016/j.bjid.2023.102758 36809849 10.1016/j.bjid.2023.102758 PMC10024133

[14] Zhen S, Zhao Y, Chen Z et al (2023) Assessment of mortality-related risk factors and effective antimicrobial regimens for treatment of bloodstream infections caused by carbapenem-resistant Pseudomonas aeruginosa in patients with hematological diseases. Front Cell Infect Microbiol 131156651. 10.3389/fcimb.2023.1156651 10.3389/fcimb.2023.1156651 PMC10320591 37415825

[15] Soares de Moraes L, Gomes Magalhaes GL, Material Soncini JG, Pelisson M, Eches Perugini MR, Vespero EC (2022) High mortality from carbapenem-resistant Klebsiella pneumoniae bloodstream infection. Microb Pathog 167:105519. 10.1016/j.micpath.2022.105519 35483557 10.1016/j.micpath.2022.105519

[16] Tang Y, Wu X, Cheng Q, Li X (2020) Inappropriate initial antimicrobial therapy for hematological malignancies patients with Gram-negative bloodstream infections. Infection 48:109–116. 10.1007/s15010-019-01370-x 31677085 10.1007/s15010-019-01370-x

[17] Guarana M, Nucci M, Nouér SA (2019) Shock and early death in hematologic patients with Febrile Neutropenia. Antimicrob Agents Chemother 63. 10.1128/aac.01250-19 10.1128/AAC.01250-19 PMC6811434 31405857

[18] Sheikh BA, Bhat BA, Mir MA (2022) Antimicrobial resistance: new insights and therapeutic implications. Appl Microbiol Biotechnol 106:6427–6440. 10.1007/s00253-022-12175-8 36121484 10.1007/s00253-022-12175-8

[19] Falcone M, Tiseo G, Carbonara S et al (2023) Mortality attributable to Bloodstream infections caused by different carbapenem-resistant gram-negative Bacilli: results from a nationwide study in Italy (ALARICO Network). Clin Infect Dis 76:2059–2069. 10.1093/cid/ciad100 36801828 10.1093/cid/ciad100

[20] Scheich S, Weber S, Reinheimer C et al (2018) Bloodstream infections with gram-negative organisms and the impact of multidrug resistance in patients with hematological malignancies. Ann Hematol 97:2225–2234. 10.1007/s00277-018-3423-5 10.1007/s00277-018-3423-5 29974230

[21] Chen J, Ma H, Huang X et al (2022) Risk factors and mortality of carbapenem-resistant Klebsiella pneumoniae bloodstream infection in a tertiary-care hospital in China: an eight-year retrospective study. Antimicrob Resist Infect Control 11:161. 10.1186/s13756-022-01204-w 10.1186/s13756-022-01204-w PMC9761986 36536423

[22] Wang S, Song Y, Shi N et al (2023) Characteristics, outcomes, and Clinical Indicators of Bloodstream Infections in neutropenic patients with hematological malignancies: a 7-Year retrospective study. Infect Drug Resist 16:4471–4487. 10.2147/idr.S413454 37449245 10.2147/IDR.S413454 PMC10337688

[23] Merdad R, Alyami A, Basalim A et al (2023) Bloodstream gram-negative bacterial infections in adult patients with leukemia: a retrospective review of medical records in a tertiary care hospital in Western Saudi Arabia. J Infect Public Health 16:1525–1530. 10.1016/j.jiph.2023.07.010 10.1016/j.jiph.2023.07.010 37557008

[24] Murali NA, Ganesan P, Vijayakumar V et al (2016) Increasing incidence of multidrug-resistant Gram-negative septicaemia during induction therapy of acute myeloid leukaemia. J Hosp Infect 93:314–315. 10.1016/j.jhin.2016.04.012 10.1016/j.jhin.2016.04.012 27206967

[25] Fupin H, Yan G, Demei Z et al (2022) CHINET surveillance of antimicrobial resistance among the bacterial isolates in 2021. Chin J Infect Chemother 22:521–530. 10.16718/j.1009-7708.2022.05.001

[26] Yahav D, Giske CG, Grāmatniece A, Abodakpi H, Tam VH, Leibovici L (2020) New β-Lactam-β-Lactamase inhibitor combinations. Clin Microbiol Rev. 10.1128/cmr.00115-20 33177185 10.1128/CMR.00115-20 PMC7667665

[27] Trecarichi EM, Pagano L, Martino B et al (2016) Bloodstream infections caused by Klebsiella pneumoniae in onco-hematological patients: clinical impact of carbapenem resistance in a multicentre prospective survey. Am J Hematol 91:1076–1081. 10.1002/ajh.24489 10.1002/ajh.24489 27428072

[28] Tacconelli E, Tumbarello M, Bertagnolio S et al (2002) Multidrug-resistant Pseudomonas aeruginosa bloodstream infections: analysis of trends in prevalence and epidemiology. Emerg Infect Dis 8:220–221. 10.3201/eid0802.010121 11897080 10.3201/eid0802.010121 PMC2732442

[29] Routsi C, Pratikaki M, Platsouka E et al (2013) Risk factors for carbapenem-resistant Gram-negative bacteremia in intensive care unit patients. Intensive Care Med 39:1253–1261. 10.1007/s00134-013-2914-z 23604133 10.1007/s00134-013-2914-z

[30] Sheng WH, Liao CH, Lauderdale TL et al (2010) A multicenter study of risk factors and outcome of hospitalized patients with infections due to carbapenem-resistant Acinetobacter baumannii. Int J Infect Dis 14:e764. 10.1016/j.ijid.2010.02.2254 20646946 10.1016/j.ijid.2010.02.2254

[31] Ferrer R, Martin-Loeches I, Phillips G et al (2014) Empiric antibiotic treatment reduces mortality in severe sepsis and septic shock from the first hour: results from a guideline-based performance improvement program. Crit Care Med 42:1749–1755. 10.1097/ccm.0000000000000330 24717459 10.1097/CCM.0000000000000330

[32] Chumbita M, Puerta-Alcalde P, Yáñez L et al (2023) High rate of Inappropriate antibiotics in patients with hematologic malignancies and Pseudomonas aeruginosa Bacteremia following International Guideline recommendations. Microbiol Spectr 11:e0067423. 10.1128/spectrum.00674-23 37367629 10.1128/spectrum.00674-23 PMC10434044

[33] Garcia-Vidal C, Cardozo-Espinola C, Puerta-Alcalde P et al (2018) Risk factors for mortality in patients with acute leukemia and bloodstream infections in the era of multiresistance. PLoS ONE 13:e0199531. 10.1371/journal.pone.0199531 29953464 10.1371/journal.pone.0199531 PMC6023133

[34] Sartori MT, Zurlo C, Bon M et al (2020) Platelet-derived microparticles bearing PF4 and Anti-GAGS immunoglobulins in patients with Sepsis. Diagnostics (Basel). 10.3390/diagnostics10090627 32846949 10.3390/diagnostics10090627 PMC7555115

[35] Li J, Feng X, Wang J et al (2023) Acinetobacter spp. bloodstream infection in hematological patients: a 10-year single-center study. BMC Infect Dis 23:796. 10.1186/s12879-023-08789-6 37964192 10.1186/s12879-023-08789-6 PMC10648370

[36] Micozzi A, Gentile G, Minotti C et al (2017) Carbapenem-resistant Klebsiella pneumoniae in high-risk haematological patients: factors favouring spread, risk factors and outcome of carbapenem-resistant Klebsiella pneumoniae bacteremias. BMC Infect Dis 17:203. 10.1186/s12879-017-2297-9 28283020 10.1186/s12879-017-2297-9 PMC5345173

[37] Chen CY, Tien FM, Sheng WH et al (2017) Clinical and microbiological characteristics of bloodstream infections among patients with haematological malignancies with and without neutropenia at a medical centre in northern Taiwan, 2008–2013. Int J Antimicrob Agents 49:272–281. 10.1016/j.ijantimicag.2016.11.009 28109554 10.1016/j.ijantimicag.2016.11.009

[38] Ma J, Li N, Liu Y et al (2017) Antimicrobial resistance patterns, clinical features, and risk factors for septic shock and death of nosocomial E coli bacteremia in adult patients with hematological disease: a monocenter retrospective study in China. Med (Baltim) 96e6959. 10.1097/md.0000000000006959 10.1097/MD.0000000000006959 PMC5457869 28538389

[39] Tang Y, Cheng Q, Yang Q et al (2018) Prognostic factors and scoring model of hematological malignancies patients with bloodstream infections. Infection 46:513–521. 10.1007/s15010-018-1151-3 29767394 10.1007/s15010-018-1151-3

[40] Zhai W, Zhang X, Wei J et al (2015) A prospective observational study of antibiotic therapy in Febrile Neutropenia patients with hematological malignances from multiple centers in Northeast China. Int J Infect Dis 37:97–103. 10.1016/j.ijid.2015.04.015 25931196 10.1016/j.ijid.2015.04.015

[41] Peri AM, Edwards F, Henden A et al (2023) Bloodstream infections in neutropenic and non-neutropenic patients with haematological malignancies: epidemiological trends and clinical outcomes in Queensland, Australia over the last 20 years. Clin Exp Med 23:4563–4573. 10.1007/s10238-023-01206-x 37815735 10.1007/s10238-023-01206-x PMC10725384

